# Textbook outcomes after oesophagectomy: a single-centre observational study

**DOI:** 10.1186/s12893-023-02253-7

**Published:** 2023-12-08

**Authors:** Vered Buchholz, Riley Hazard, Dong-Kyu Lee, David S Liu, Wendell Zhang, Sharon Chen, Ahmed Aly, Stephen Barnett, Peter Le, Laurence Weinberg

**Affiliations:** 1 Department of Surgery, Austin Health, University of Melbourne, Heidelberg, Melbourne, Australia; 2 Department of Anesthesia, Austin Health, Melbourne, VIC Australia; 3 Department of Anesthesiology and Pain Medicine, Dongguk University Ilsan Hospital, Goyang, Republic of Korea; 4 General and Gastrointestinal Surgery Research and Trials Group, The University of Melbourne, Austin, USA; 5 Division of Cancer Surgery, The Peter MacCallum Cancer Centre, Melbourne, Precinct, VIC Australia; 6 Department of Critical Care, University of Melbourne, Victoria, Australia

**Keywords:** Oesophagectomy, Cancer, Surgery, Costs, Complications, Anaesthesia, Textbook outcome

## Abstract

**Background:**

Textbook outcomes is a composite quality assurance tool assessing the ideal perioperative and postoperative course as a unified measure. Currently, its definition and application in the context of oesophagectomy in Australia is unknown. The aim of this study was to assess the textbook outcomes after oesophagectomy in a single referral centre of Australia and investigate the association between textbook outcomes and patient, tumour, and treatment characteristics.

**Methods:**

An observational study was retrospectively performed on patients undergoing open, laparoscopic, or hybrid oesophagectomy between January 2010 and December 2019 in a single cancer referral centre. A textbook outcome was defined as the fulfillment of 10 criteria: R0 resection, retrieval of at least 15 lymph nodes, no intraoperative complications, no postoperative complications greater than Clavien-Dindo grade III, no anastomotic leak, no readmission to the ICU, no hospital stay beyond 21 days, no mortality within 90 days, no readmission related to the surgical procedure within 30 days from admission and no reintervention related to the surgical procedure. The proportion of patients who met each criterion for textbook outcome was calculated and compared. Selected patient-related parameters (age, gender, BMI, ASA score, CCI score), tumour-related factors (tumour location, tumour histology, AJCC clinical T and N stage and treatment-related factor [neoadjuvant chemotherapy and surgical approach]) were assessed. Disease recurrence and one year survival were also evaluated.

**Results:**

110 patients who underwent oesophagectomy were included. The overall textbook outcome rate was 24%. The difference in rates across the years was not statistically significant. The most achieved textbook outcome parameters were ‘no mortality in 90 days’ (96%) and ‘R0 resection’ (89%). The least frequently met textbook outcome parameter was ‘no severe postoperative complications’ (58%), followed by ‘no hospital stays over 21 days’ (61%). No significant association was found between patient, tumour and treatment characteristics and the rate of textbook outcome. Tumour recurrence rate and overall long term survival was similar between textbook outcome and non-textbook outcome groups. Patients with R0 resection, no intraoperative complication and a hospital stay less than 21 days had reduced mortality rates.

**Conclusions:**

Textbook outcome is a clinically relevant indicator and was achieved in 24% of patients. Severe complications and a prolonged hospital stay were the key criteria that limited the achievement of a textbook outcome. These findings provide meticulous evaluation of oesophagectomy perioperative care and provide a direction for the utilisation of this concept in identifying and improving surgical and oncological care across multiple healthcare levels.

## Background

Quality of care is increasingly scrutinised across many national health systems. The demand for transparency, continuous monitoring and ongoing improvement originates at multiple levels—patients choosing a care provider, individual hospitals looking to improve their care, professional societies seeking to benchmark and standardise treatment across services and stakeholders defining resource allocation [1]. Oesophagectomy, the mainstay of oesophageal cancer care, attracts particular attention in that context, given its substantial morbidity rate and associated high costs [2–5].

Quality assessments commonly use individual parameters, such as complications and mortality rate, length of stay and readmission rate. However, those merits cover limited aspects of the oesophagectomy perioperative pathway and do not amount to a standardised comparative tool [6–8]. A textbook outcome is a comprehensive measure comprising short-term variables reflecting an ideal perioperative course. It was first introduced in the Netherlands for colon cancer resections in 2010 and later adapted for gastroesophageal cancer surgery by Busweiler and colleagues in 2017 [9, 10].

Recently, an international consensus updated the textbook outcome quality measure parameters, adding further specificity for oesophageal surgery [11]. The literature on oesophagectomy textbook outcome remains scarce and mainly originates from the Netherlands and the USA [12–15], with a single international cohort study covering 10 months across 41 countries [16]. Most published data are registry-based, and variations in the textbook outcome components’ definitions compromise comparison between studies.

The current literature shows that this unforgiving ‘all-or-none’ tool can only be realised in 30% [10, 12] to 40% of patients [14, 16]. Achieving a textbook outcome was linked to long-term benefits, such as increased disease-free and overall survival [12, 13, 17], signifying its importance beyond short-term performance monitoring. The objectives of this study were to explore the rates of the textbook outcome at a local level in an Australian cancer centre, explore potential patients’ tumour and treatment predictors of textbook outcome and investigate their possible association with long-term oncological benefits.

## Methods

### Setting

This study was conducted at Austin Health, a university-affiliated tertiary referral centre for oesophago-gastric cancer care and is reported by the Strengthening the Reporting of Observational Studies in Epidemiology (STROBE) guidelines for observational studies [18, 19].

### Participants

All adult patients who underwent open, laparoscopic or hybrid two- or three-stage oesophagectomy between January 2010 and December 2019 were included in the study. Patients were identified using the International Statistical Classification of Diseases and Related Health Problems 10th Revision (ICD-10) and codes specific to oesophagectomy. Two surgical units, the upper gastrointestinal and thoracic surgical units, performed all the surgical procedures.

### Neoadjuvant and surgical treatment

Prior to surgery, eligible patients received either neoadjuvant chemotherapy (Epirubicin, Cisplatin, and fluorouracil or Fluorouracil, leucovorin, oxaliplatin and Docetaxel from 2018) or chemoradiotherapy (carboplatin, paclitaxel with 41.4 Gy). Transthoracic oesophagectomy was performed 6–10 weeks after completion of the neoadjuvant course, with thoracic or cervical anastomosis.

### Preoperative optimisation and perioperative care

All patients underwent an enhanced recovery after surgery (ERAS) program aligned with international guidelines, including a comprehensive pre-optimisation program for smoking and alcohol cessation [20]. All participants underwent a comprehensive multidisciplinary assessment, with optimisation of nutrition, medical comorbidities, and haemoglobin levels, based on the National Blood Authority of Australia’s Patient Blood Management Initiative [21]. General anaesthesia was managed using an ERAS protocol designed to standardise care. Postoperatively, all patients were admitted to the intensive care unit (ICU) for at least one overnight stay and discharged to a dedicated surgical ward. The ERAS protocol was applied throughout the admission.

### Data collection

All data were sourced directly by the authors using prospectively recorded patients’ variables from the hospital’s electronic health records (Cerner® Millennium, Kansas, USA). Preoperative patient parameters included demographic information, body mass index (BMI), history of smoking and alcohol abuse, the American Society of Anaesthesiologists Society (ASA) score, comorbidities, Charlson Comorbidity Index (CCI) score, history of previous abdominal or thoracic surgery, preoperative blood values, pathological diagnosis and neoadjuvant treatment. Intraoperative parameters included type of surgery (open or minimally invasive), surgical approach (transthoracic or transhiatal), operative time, volumes of transfused crystalloids, colloids and blood products, use of vasoactive medications and intraoperative complications. Postoperative variables included ICU admission and care duration, postoperative blood values, blood products transfusion, tumour histology, location and stage as per the American Joint Committee on Cancer (AJCC) 8th edition, postoperative complications, length of hospital stay, discharge destination, readmissions, tumour recurrence times and mortality (30 days, 90 days, 1 year and overall).

### Textbook outcome parameters

The primary outcome was the rate of textbook outcomes. A textbook outcome was achieved when all the following 10 criteria were met: R0 resection, retrieval of at least 15 lymph nodes, no intraoperative complications, no postoperative complications greater than Clavien-Dindo grade III, no anastomotic leak, no readmission to the ICU, no hospital stay beyond 21 days, no mortality within 90 days, no readmission related to the surgical procedure within 30 days from admission and no reintervention (reoperation, endoscopic or radiologic) related to the surgical procedure. Anastomotic leaks were classified per the international Esophagectomy Complications Consensus Group (ECCG) definitions [22].

### Statistical analysis

Statistical analysis was performed using the R software (version 4.2.1; 2022, R Core Team, Vienna, Austria). The Mann–Whitney U test was used for comparing continuous variables, and Fisher’s exact test was used for comparing categorical variables. Data are presented as mean ± standard deviation (SD), median (interquartile range [minimum : maximum]) or number (percentile). Calculated odds ratios (OR) were provided with 95% confidence intervals (CI). The proportion of patients who met each criterion for textbook outcome was calculated and compared across the years. A selection of patient-related parameters (age, gender, BMI, ASA score, CCI score), tumour-related factors (tumour location, tumour histology, AJCC clinical T and N stage and treatment-related factor [neoadjuvant chemotherapy and surgical approach]) were assessed.

Disease recurrence and long-term survival (1 year and overall) were also evaluated. A multivariate logistic regression model was then used to examine the impact of patient, tumour or treatment variables on the textbook outcome. A Kaplan–Meier survival curve with the log-rank test was used to investigate the survival of patients with and without a textbook outcome. Multivariate Cox regression models were used to study the relationship between each textbook outcome criterion and survival time. No missing values were imputed. Statistical significance was defined as a two-tailed *p*-value < 0.05.

## Results

**Fig. 1:**
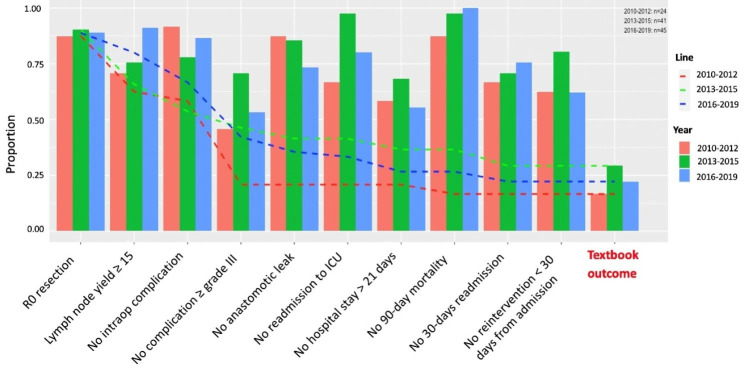
Proportion of patients who fulfilled individual textbook outcome parameters over three time periods. The cumulative proportions of patients achieving textbook outcome are plotted as dashed lines. (R0: negative margin resection; ICU: intensive care unit)

Patients (n = 110) who underwent oesophagectomy were included in the study cohort and the overall textbook outcome rate was 24% (26 patients). The rate of textbook outcome variated over the years and was the highest during 2013–2015 at 29% and lowest during 2010–2012 at 17%. However, the difference in textbook outcome rates across the years was not statistically significant (Fig. 1). The most achieved textbook outcome parameters were ‘no mortality in 90 days’ (96%) and ‘R0 resection’ (89%). The least frequently met textbook outcome parameter was ‘no severe postoperative complications’ (58%), followed by ‘no hospital stays over 21 days’ (61%) (Fig. 1 and Table 1). The upper gastrointestinal unit operated on 85 (77.3%) patients and the thoracic unit on 25 (22.7%) patients. Textbook outcomes for patients operated on by the gastrointestinal unit was 28% vs. 16% for the thoracic unit (p = 0.059; 95%CI: 1.000 to 3.979).

**Table 1:** Textbook outcome for patients undergoing oesophagectomy

Textbook outcome criteria	Total cohort (n = 110)
R0 resection	98 (89%)
Lymph node yield ≥ 15	89 (81%)
No intraoperative complication	93 (85%)
No complication ≥ grade III	64 (58%)
No anastomotic leak	89 (81%)
No readmission to ICU	92 (84%)
No hospital stay > 21 days	67 (61%)
No 90-day mortality	106 (96%)
No 30-days readmission	79 (72%)
No reintervention < 30 days from admission	76 (69%)
**Total number of patients with a textbook outcome**	**26 (24%)**

Data presented as number (proportion)

Forty-six patients (42%) did not experience a textbook outcome due to severe complications (CD grade > IIIa). The most common severe complications category observed in those patients was pulmonary (30%, n = 33), followed by gastrointestinal (14%, n = 15). Twenty patients (18%) experienced an anastomotic leak, of whom nine patients required intervention or escalation of treatment (CD IIIa to IVb) and none died (Table 2).

**Table 2:** Complications profile for patients without a textbook outcome

COMPLICATIONS	Total (n = 110)
**Total oesophagectomy key complications**	37 (34%)
Severe complications category	
Gastrointestinal	15 (14%)
Cardiovascular	3 (3%)
Pulmonary	33 (30%)
Infection	5 (5%)
Metabolic	1 (1%)
Neurologic	5 (5%)
Psychiatric	1 (1%)
Other	8 (7%)

Oesophagectomy key complications: anastomotic leak, conduit necrosis, chyle leak and laryngeal nerve palsy (categorised as per the ECCG definitions, any grade)

Data presented as number and proportion

### Textbook outcome correlation with the patient, tumour and treatment factors

The patient, oncological and operative characteristics considered for analysis for patients with or without textbook outcome are summarised in Table 3. The study population was predominantly male (83%, n = 93), with a mean age of 64.5 years (± 9.7). The mean CCI score was 4.4 (± 1.6), most patients had an ASA score of III or IV and the mean BMI was 27 (± 4.9). The prevalent tumour histopathology was adenocarcinoma (82%, n = 92) and most tumours were in the gastroesophageal junction or distal oesophagus (84%, n = 93). Most patients received neoadjuvant therapy (70%, n = 77) and four (3.6%) patients were operated on following incomplete response to definite chemoradiotherapy.

**Table 3:** Patient, tumour and treatment characteristics of the study population. Data are presented for patients with or without textbook outcome

		Total (n = 110)	No textbook outcome (n = 84)	Textbook outcome (n = 26)	*p*-value
Patient variables					
**Age (years)**	Mean ± SD	64.5 ± 9.7	64.8 ± 9.5	63.5 ± 10.4	0.418
**Sex (male)**	n (%)	91 (83%)	70 (83%)	21 (81%)	0.771
**BMI (kg/m** ^**2**^ **)**	Mean ± SD	27.0 ± 4.9	27.0 ± 4.9	27.0 ± 5.2	0.933
**ASA grade**	n (%)				
ASA I–II	n (%)	37 (34%)	26 (31%)	11 (42%)	0.344
ASA III–IV	n (%)	73 (66%)	58 (69%)	15 (58%)	
**CCI score**	Mean ± SD	4.4 ± 1.6	4.5 ± 1.6	4.1 ± 1.4	0.24
**Tumour variables**					
**Tumour location**					
Proximal third	n (%)	3/108 (3%)	2/83 (2%)	1/25 (4%)	0.514
Middle third	n (%)	5/108 (5%)	5/83 (6%)	0/25 (0%)	
Distal third	n (%)	34/108 (31%)	28/83 (34%)	6/25 (24%)	
Gastroesophageal junction	n (%)	59/108 (55%)	42/83 (51%)	17/25 (68%)	
Other	n (%)	7/108 (6%)	6/83 (7%)	1/25 (4%)	
**Tumour histological type**					
Adenocarcinoma	n (%)	90 (82%)	67 (80%)	23 (88%)	0.751
Squamous cell carcinoma	n (%)	13 (12%)	11 (13%)	2 (8%)	
Other	n (%)	7 (6%)	6 (7%)	1 (4%)	
**cT-stage**					
T0–2, n (%)		16/51 (31%)	12/41 (29%)	4/10 (40%)	0.705
T3–4, n (%)		35/51 (69%)	29/41 (71%)	6/10 (60%)	
**cN-stage**					
N0, n (%)		64/104 (62%)	46/79 (58%)	18/25 (72%)	0.196
N1, n (%)		38/104 (37%)	32/79 (41%)	6/25 (24%)	
N2–3, n (%)		2/104 (2%)	1/79 (1%)	1/25 (4%)	
**Treatment characteristics**					
**Neoadjuvant therapy**					
None		7 (6%)	4 (5%)	3 (12%)	0.704
Chemotherapy		70 (64%)	54 (64%)	16 (62%)	
Chemoradiotherapy		6 (5%)	5 (6%)	1 (4%)	
Other		27 (25%)	21 (25%)	6 (23%)	
**Surgical approach**					
Open		94 (85%)	73 (87%)	21 (81%)	0.672
Minimally invasive		3 (3%)	3 (3%)	0 (0%)	
Hybrid thoracoscopy		13 (12%)	8/83 (10%)	5 (19%)	

BMI, Body Mass Index; ASA, American Society of Anaesthesiology; CCI, Charlson Comorbidity Index

In total, 94 patients (85.4%) underwent open surgery, 13 (2.7%) patients underwent a hybrid procedure and three patients (2.7%) had minimally invasive oesophagectomy. The predominant approach was the two-stage oesophagectomy (61.8%). Multivariate logistic regression was applied to patient, tumour and treatment characteristics. No significant association was found between the selected parameters and the rate of textbook outcome.

### Recurrence and survival

Tumour recurrence was observed in 43 patients (39%) overall. The tumour recurrence rate was similar in both groups: 38.5% (10/26) for patients with textbook outcomes and 39.3% (33/84) without textbook outcomes. Postoperative mortality rates were low. One patient died within 30 days of the operation, four patients (3.6%) died within the first 90 days after the operation and 16 patients (14.5%) died within the first year of their operation. Overall, 40 patients had documented death since their operation. However, 45 patients were lost for long-term follow-up. Using overall mortality for patients with complete follow-up (n = 65), the median survival was 2.2 years for all patients, 2.8 years for patients with textbook outcomes and 2 years for patients without textbook outcomes.

**Fig. 2:**
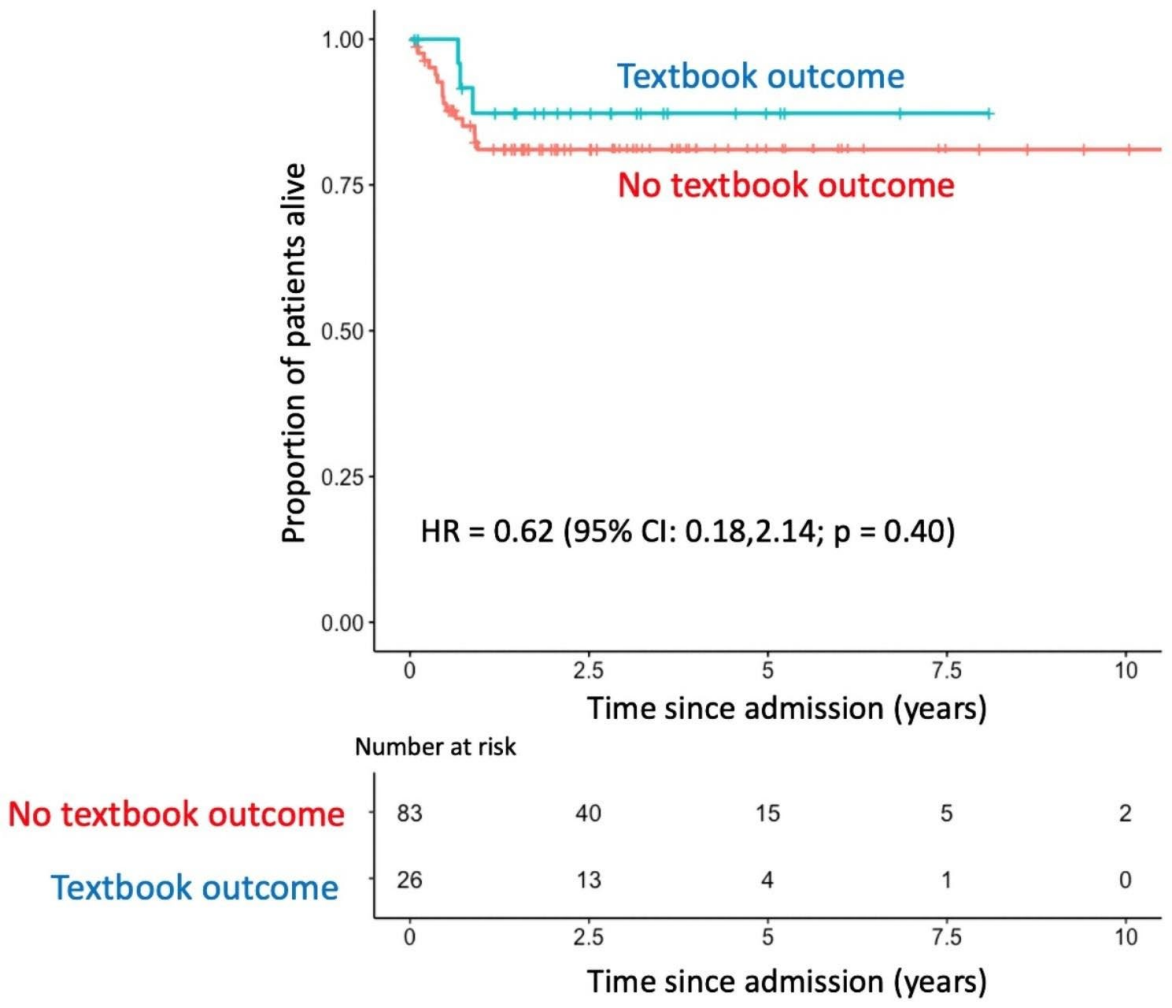
Kaplan–Meier survival curve for patients with and without a textbook outcome

**Fig. 3:**
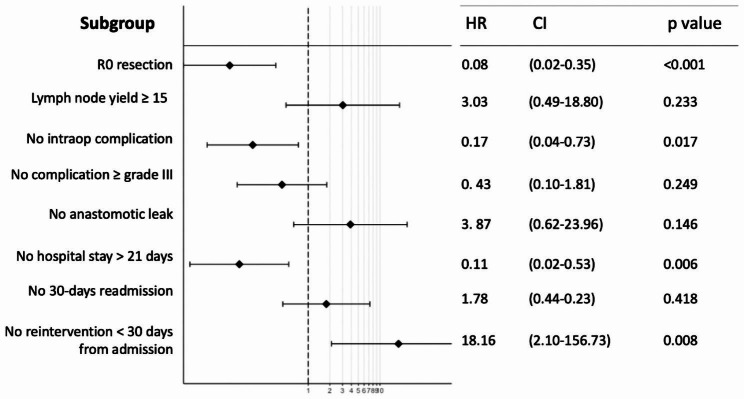
Multivariate analysis showing the impact of each criterion of the textbook outcome on 1-year survival. Results are presented as hazard ratios (HR) with 95% confidence (CI)

Kaplan–Meier overall survival curves (Fig. 2) did not demonstrate a survival difference between patients with and without a textbook outcome (HR: 0.62, 95% CI: 0.18–2.14; *p* = 0.4). A multivariate model was generated for each criterion of the textbook outcome to investigate their effect on survival (Fig. 3). Patients with R0 resection, no intraoperative complication and a hospital stay shorter than 21 days demonstrated reduced mortality rates (*p* < 0.001, *p* = 0.017, *p* = 0.006). Patients who had a reintervention within 30 days since the oesophagectomy showed increased mortality rates (*p* = 0.008). Other criteria of the textbook outcome were not associated with survival time. Readmission to the ICU and 90-day mortality were removed from the model due to the convergence of the log-likelihood.

## Discussion

In this study, we investigated the use of textbook outcome, a composite quality assurance tool, to assess perioperative care for patients undergoing oesophagectomy in a single cancer referral centre. A textbook outcome was achieved in 24% of the patients. Postoperative complications of CD grade III and above and a prolonged hospital stay of more than 21 days, which are strongly linked, were the limiting criteria from achieving a textbook outcome.

Our textbook outcome variables were based on the recently published Oesophago-Gastric Anastomotic Audit (OGAA) collaborative international cohort study [16] and the updated international consensus for textbook outcome criteria [11]. The revised criteria omitted subjective criteria, such as ‘complete resection judged by the surgeon’, increased the complications severity threshold to Clavien-Dindo grade III [11, 16] and extended the postoperative mortality from 30 to 90 days [16]. Additionally, in accordance with the international updated international consensus, we included the oesophagectomy-specific criterion ‘no leakage of all ECCG grades’ [22]. This augments the value and relevance of this quality indicator in the context of oesophageal surgery [11]. Our lower textbook outcome rate of (24%) may reflect our choice of stricter criteria.

Previous multicentre studies showed an association between centre-level factors and the rate of textbook outcomes. The OGAA audit demonstrated that high-income country centres, with a daily on-call rota of oesophago-gastric surgeons, radiology and the application of ERAS protocol, achieve significantly better textbook outcomes [16]. Textbook outcomes were also directly related to centre volume; considerably higher rates were demonstrated for hospitals performing a high volume of cases per year, where high volume was defined as more than 40 [13] or 50 cases per year [16]. Our study data were derived from a low- to medium-volume centre, and our textbook outcome rates are similar to those reported for similar-sized centres in the Van der Werf series: 15% in low-volume centres (0–19 cases) and 21% in medium-volume centres (20–39 cases) [13]. However, our textbook outcome rates were lower than the 38.5% achieved in low-volume centres (< 28 cases), as reported in the OGAA study [16].

The number of oesophagectomies performed at our institution increased between 2010 and 2012 (n = 24 or 8 per year) and 2013–2015 (n = 41 or 14 per year). Further, 45 consequent patients were operated on between the following four years (2016–2019 or 11 per year), implying that fewer procedures were performed than in the previous period. Although these may seem to be minor yearly variations, this may partly explain the better outcomes observed in our study during the 2013–2015 period. Aligned with the above, increased surgical volume has been strongly linked to a reduced complications (rate and severity) when comparing high-volume units to low-volume units [23], or high-volume surgeons to low-volume surgeons within a high-volume unit [24].

While the retrieval of more than 15 lymph nodes was the limiting criterion in previous studies [10, 13], complications severity was the prominent cause for failure to achieve textbook outcome in the OGAA multicentre cohort [16]. The findings from our study support this, despite altering the definition of severe complications from greater than CD grade II [10, 12, 13] to above CD grade III. Similar to the OGAA study, all our data were sourced directly from patients’ files. This more likely reflects a ‘real-life’ account compared with previous registry bases analyses [10, 12, 15], making complications rate the major hurdle for textbook outcome. Our cohort had a high incidence of severe complications. Therefore, the rate of patients achieving the criterion ‘no severe complications of CD grade III and above’ in our cohort was only 58% compared to 74.7% in the OGAA international multicentre cohort, which used a similar set of textbook criteria. Complications rate is not only a short-term quality assessment measure but also linked to increased costs [5, 25] and reduced long-term survival [26, 27]. Therefore, a closer exploration into measures to reduce severe complication rates is warranted.

The high rates of pulmonary complications in our series may be reflective of most patients (85%) undergoing an open approach. Several randomised studies have demonstrated the benefit of minimally invasive esophagectomy compared to the open approach in reducing complications [16, 26]. However, the association between a thoracoscopic approach and improved textbook outcomes or a reduced complication rate is conflicting. In a large population-based study of 1727 patients undergoing open esophagectomy or minimally invasive oesophagectomy, mortality and pulmonary complications were similar between the groups, however, anastomotic leaks and reinterventions were more frequently observed after a minimally invasive approach [28]. The OGAA series reported marginal but statistically significant improvements in textbook outcomes with minimally invasive oesophagectomy [16]. However, the transhiatal approach failed to demonstrate significant outcome benefits, which may reflect a patient selection bias. Busweiler et al. linked better textbook outcomes to a minimally invasive approach [10], while Van Der Kaajj et al. did not observe significant benefits with a minimally invasive approach over the traditional open approach [14]. Finally, in the series of minimally invasive oesophagectomies reported by Xu et al., 46% of the patients had severe complications [17], which is similar to the complication rate reported in our series. Our findings suggest that the thoracoscopic approach is one of the multiple factors that may reduce the severity of complications, particularly respiratory complications, however, if applied, should be combined with other measures such as respiratory prehabilitation, ERAS protocol application, early mobilisation, and judicious perioperative fluid management.

Specific patient, disease and treatment factors were linked to textbook outcome in the existing literature. Male gender, older age, high CCI score, ASA score and BMI, use of preoperative enteral nutrition and squamous cell carcinoma correlated with decreased textbook outcome rates [10, 12, 16]. Pathological AJCC TNM stage and neoadjuvant treatment did not correlate with textbook outcomes in the Van der Kaaij single-centre analysis [14] or the OGAA multicentre study [16] when multivariate analysis was applied. The latter study also demonstrated that anastomosis above the azygos and a minimally invasive approach increased the likelihood of textbook outcome. We were unable to establish a correlation with any of the perioperative factors examined. Our patients’ cohort perioperative characteristics differ from preceding studies in their morbidity indexes and surgical approach [10, 12, 16]: 73% of our patients had an ASA score > 3, the mean CCI score was > 4 in both patient groups and the predominant approach was open, in comparison to better morbidity scores and prevalence of minimally invasive approach. Moreover, our small size cohort may have prevented us from reaching statistically significant results.

The textbook outcome comprises key surgical and oncological outcomes representing the ideal short-term postoperative course. The clinical relevance of this quality assurance tool is intensified if linked to the principal oncological outcome measure: long-term disease-free and overall survival. Individual textbook outcome criteria, such as R0 resection and high lymph node yield, were previously recognised as survival predictors [29]. Our results demonstrated an association between three of the 10 criteria: R0 resection, no intraoperative complications, length of stay < 21 days and long-term survival. However, we could not demonstrate a survival benefit for patients who achieved a textbook outcome over patients who did not. Nevertheless, Kalff et al. showed that patients with textbook outcome benefitted from 17 months of disease-free survival and 22 months of overall survival [12]. This was consistent with a 17-month overall survival benefit highlighted by the Kulshrestha et al. group [15]. Similarly, van der Werf et al. reported an increased survival rate among patients with textbook outcome [8]. Our sample size and a significant loss for follow-up may have compromised our survival analysis and our ability to reflect a similar advantage.

The textbook outcome is a unique measure with multilevel relevance: it encourages improvement in patients’ short- and long-term outcomes, enhances the individual hospital quality of care and costs reduction [30] and assists the healthcare system in unifying care standards across services. To date, only a few studies have explored the application of textbook outcome in the context of oesophagectomy, none of them in Australia. Our study adds to this limited body of data by using international benchmarking definitions and an updated set of textbook outcome parameters that include relevant oesophagectomy-specific criteria [11, 22]. We present real-life single-centre data sourced directly from patient files and meticulously reviewed. As such, it provides a reliable short-circuit feedback tool for reviewing and improving the quality of care locally and guiding resource allocation. A robust dataset can be established to augment the existing national quality control of oesophageal cancer care if applied nationally.

The limitations of our study include its small cohort size and single-centre setup. Whilst the proportion of patients who fulfilled individual textbook outcome parameters was higher over the 2013 to 2015 period, this was not statistically significant. This may reflect the small cohort size, variability in the number of patients who underwent oesophagectomy, and variations in surgeons’ yearly procedure volume. Moreover, during this period, there were no significant difference observed in the number of complications. Similarly, the non-significant differences observed in textbook outcomes between the upper gastrointestinal and thoracic units may be related to differences in patient complexity, disease severity and treatment factors. We acknowledge there may be also variations in practice between the different surgical units and even between individual surgeons that affect outcomes. These inherent confounders are difficult to quantify. The textbook outcome criteria used in our study, though an updated version, differ from most earlier studies. This hampers comparison with their results. Our long-term outcome analysis was compromised by loss to follow-up, which may have limited our long-term outcome analysis. Lastly, the financial implications of textbook outcome were not explored and are an area for further study.

## Conclusion

In conclusion, we used the textbook outcome composite measure to evaluate our hospital performance, create a feedback circuit and help direct further policies and resource allocation to improve our cancer care. The textbook outcome is an ideal ‘all-or-none’ tool, which is difficult to achieve, and was realised in one of four patients. We demonstrated that mitigation of complication severity is the least achieved criterion and warrants particular attention in the context of oesophagectomy perioperative care. Although we could not identify predictors for the textbook outcome or survival benefits for patients with the textbook outcome, our study provided a transparent report of our centre’s results that benefits patients, care providers and stakeholders alike. Expansion of its use will promote excellence of care across the health system.

## Data Availability

The database used and analysed during this study is available from the corresponding author on reasonable request.

## Abbreviations

BMI: Body mass index
ICU: Intensive Care Unit
ASA: American Society of Anaesthesiologists Society
CCI: Charlson Comorbidity Index
OGAA: Oesophago-Gastric Anastomotic Audit
ECCG: Esophagectomy Complications Consensus Group
